# Spotted Fever Group Rickettsioses among Hospitalized Patients and Circulation of *Rickettsia* in Ticks, Kazakhstan, 2019

**DOI:** 10.3201/eid3110.250037

**Published:** 2025-10

**Authors:** Yekaterina V. Bumburidi, Dmitriy V. Berezovskiy, Bakhytkul T. Zhakipbayeva, Roberta Z. Horth, Alexander J. Millman, William L. Nicholson, Galina E. Zemtsova, Victoria Seffren, Elina R. Maltseva, Zhanna A. Berdygulova, Yekaterina O. Ostapchuk, Yuliya V. Perfilyeva, Andrey M. Dmitrovskiy, Zhansulu K. Dukayeva, Zhanna Zh. Shapiyeva

**Affiliations:** US Centers for Disease Control and Prevention, Central Asia Office, Almaty, Kazakhstan (Y.V. Bumburidi, D.V. Berezovskiy, B.T. Zhakipbayeva, R.Z. Horth, A.J. Millman); Centers for Disease Control and Prevention, Atlanta, Georgia, USA (W.L. Nicholson, G.E. Zemtsova, V. Seffren); Almaty Branch of the National Center for Biotechnology, Almaty (E.R. Maltseva, Z.A. Berdygulova, Y.O. Ostapchuk, Y.V. Perfilyeva, A.M. Dmitrovskiy); Pavlodar Regional Hospital named after Sultanov, Pavlodar, Kazakhstan (Z.K. Dukayeva); Scientific-Practical Center for Sanitary-Epidemiological Expertise and Monitoring, National Center for Public Healthcare, Ministry of Health, Almaty (Z.Z. Shapiyeva)

**Keywords:** vector-borne infections, bacteria, zoonoses, Spotted fever group rickettsioses, Spotted fever group rickettsia, *Rickettsia sibirica*, *Rickettsia raoultii*, *Rickettsia slovaca*, rickettsioses, Kazakhstan

## Abstract

Testing for spotted fever group rickettsioses (SFGR) and the criteria for identifying suspected patients are not routinely used in Kazakhstan. In 2019, we performed a cross-sectional study in 6 sentinel hospitals in the Pavlodar region. We tested 105 hospitalized patients with SFGR-like symptoms by using PCR or indirect immunofluorescence antibody assay and identified 62 cases of SFGR. Most (78%) cases of disease were caused by *Rickettsia sibirica* and *R. raoultii*. Cutaneous signs (eschar or rash) were found in 87% of SFGR patients; 79% had a rash, 48% had an eschar, and 13% had neither. Testing of suspected rickettsia cases resulted in a 27% increase in laboratory-detected SFGR over the mean of the previous 3 years (62 vs. 49). Broadening the case definition by including fever, headache, or myalgia and expanding routine testing for suspected cases of SFGR could contribute to improved case detection and earlier treatment.

Spotted fever group rickettsioses (SFGR) are tickborne diseases that are typically characterized by nonspecific symptoms such as fever, headache, and muscle pain caused by closely related bacteria. A rash or eschar (a black scab) is present in some but not all infections, although manifestations vary depending on the rickettsia species. That variability in clinical manifestations creates challenges for diagnosing SFGR. SFGR are treatable; however, untreated SFGR cases can result in severe complications and death in otherwise healthy persons. The severity of rickettsiosis depends on the *Rickettsia* species with which the patient is infected (*1*–*3*). 

Tickborne rickettsial bacteria cannot survive on their own in the environment; they depend on a complex lifecycle involving small animal hosts, such as rodents, and can be transmitted transovarially, with ticks serving as both the primary vector and reservoirs. Infected ticks transmit spotted fever group *Rickettsia* to humans through bites. Although SFGR are globally distributed, the actual burden of illness remains unknown because of underrecognition, limited testing, and underreporting. The geographic expansion and increase in cases of SFGR worldwide can be attributed to increased human–animal contact resulting from land-use changes, climate variations, improved clinical awareness of tickborne diseases, and the increased availability of novel molecular diagnostic techniques (*4*–*7*). 

Rickettsial diseases are notifiable in Kazakhstan. By the end of 2019, SFGR were recognized as endemic in 4 regions (East Kazakhstan, Kyzylorda, Pavlodar, and North Kazakhstan). Several different *Rickettsia* species have been identified in ticks in Kazakhstan, including *R. sibirica*, *R. raoultii*, *R. slovaca*, *R. aeschlimannii*–like organism, and *R. conorii* (*8*–*12*). In addition, *R. sibirica*, *R. rauoltii*, and *R. heilongjiangensis* were identified in Russia in a region bordering the Pavlodar oblast in Kazakhstan (*13*,*14*).

The Pavlodar region has the second highest reported rate of rickettsioses in Kazakhstan. Unlike Kyzylorda, the region with the highest reported burden, no studies have documented circulating rickettsial diseases in Pavlodar (*15*). On the basis of data available in the national surveillance system (Infectious Diseases in the Republic of Kazakhstan), during 2016–2018 the Pavlodar region reported 146 clinically identified cases of SFGR. Because SFGR signs and symptoms can be nonspecific and routine laboratory confirmation is not available in Kazakhstan, cases are often missed or misdiagnosed. Therefore, the true burden of SFGR is likely higher than reported. We assessed the clinical and epidemiologic characteristics of the disease in humans (including disease burden, clinical features, and etiologic agent) and the molecular identification of *Rickettsia* species in ticks collected from the study region.

## Methods

### Study Design

We conducted a cross-sectional study during April–October 2019 among persons hospitalized with signs and symptoms consistent with SFGR. We selected 2 regional infectious disease hospitals in the city of Pavlodar (including a pediatric hospital), which serve as referral hospitals, and 4 district hospitals that reported rickettsial disease cases in 2016–2018. Participants >6 years of age were recruited during April–October, the period when the risk for seasonal tickborne transmission was highest on the basis of active surveillance for ticks in the region.

### Enrollment and Inclusion Criteria

Participants were persons hospitalized during the study period with symptoms consistent with *Rickettsia*-like illnesses (suspected cases of rickettsiosis), such as fever (>38°C) and >1 of the following clinical signs or symptoms: rash, eschar, headache, myalgia, anemia, thrombocytopenia, hepatic transaminase elevation, or clinician suspicions of rickettsiosis (*16*). Participants provided written informed consent; for persons <18 years of age, the primary caregiver consented. We excluded persons who were not residents of the Pavlodar region (residence was defined as having lived <2 weeks outside the Pavlodar region during the study period before disease onset) and children <6 years of age.

### Data Collection

Clinicians at the sentinel sites were trained to collect data at the time of patient’s inclusion to apply the standard case definition of *Rickettsia*-like illness and to notify the health department of new suspected cases of rickettsiosis. Upon receiving notifications, trained surveillance officers visited the hospitals and obtained written informed consent from hospitalized patients or their primary caregivers to participate in the study. Surveillance officers completed paper case investigation forms containing demographic, clinical, laboratory, and behavioral data. They also abstracted clinical and laboratory data from the medical and laboratory records and entered data into EpiInfo 7.2 (https://www.cdc.gov/epiinfo).

### Specimen Collection

We collected acute-phase samples, including rash or eschar biopsies or swab specimens and serum samples, at admission (*17*,*18*). We collected second serum samples (convalescent) 2–3 weeks after admission from patients with no rash or eschar or whose rash or eschar samples were PCR negative. We collected second serum samples at the hospital before discharge; however, if duration of hospitalization was <2 weeks, we invited patients by phone to the territorial polyclinic for specimen collection. We collected rash and eschar biopsies and swab samples under sterile conditions.

We stored specimens in refrigerators (4°C) at the hospital for up to 24 hours before being transported to the Pavlodar regional laboratory for PCR testing (rash and eschar samples) and freezing (at −20°C). Samples were subsequently sent to the Central Reference Laboratory for sequencing.

### Laboratory Testing of Human Specimens

#### PCR Testing and Sequencing

We extracted DNA from rash and eschar specimens using the QIAGEN DNA Mini Kit (QIAGEN, https://www.qiagen.com) according to the manufacturer’s instructions. We used a real-time genus-specific *Rickettsia* (pan-*Rickettsia*) assay targeting the 23S rRNA as a screening assay (*19*). We further tested specimens positive for rickettsial DNA in the pan-*Rickettsia* assay by using a seminested PCR to amplify and subsequently sequence a fragment of the gene encoding the protein rOmpA, appropriate for differentiation of 21 *Rickettsia* species, including the most commonly reported species circulating in the investigated region (*20*). We used the human housekeeping glyceraldehyde 3-phosphate dehydrogenase (GAPDH) gene, yielding a 157-bp fragment as an internal control (*21*). We reported PCR test results to the hospital within 7 days.

We performed elution of DNA fragments from the agarose gel using a QIAquick gel extraction kit (QIAGEN). We then sequenced the purified DNA fragments in both directions using a BigDye Terminator v3.1 Cycle Sequencing Kit and an ABI 3500XL genetic analyzer (Thermo Fisher Scientific, https://www.thermofisher.com).

Individual gene sequences underwent multiple sequence alignment using the MUSCLE algorithm in the MEGA X program (*22*). We constructed a maximum-likelihood phylogenetic tree using *Rickettsia* species from various phylogenetic groups as references. We used nonparametric bootstrapping with 500 replicates to calculate confidence estimates.

### Indirect Immunofluorescence Antibody Assay for Serum Samples

We tested serum samples using an indirect immunofluorescence antibody assay (IFA). We prepared multiantigen slides with strains of *R. raoultii*, *R. sibirica*, and *R. slovaca*. We used serum containing human IgG to spotted fever group *Rickettsia* as positive controls. Slides and positive controls were provided by the Centers for Disease Control and Prevention (CDC; Atlanta, GA, USA). For determination of acute case status, positive samples were titrated to determine endpoint titer and to examine 4-fold titer increases. Comparison of titers provided presumptive etiology for this study (*23*). We defined in IFA the species of *Rickettsia* implicated in the disease for patients with paired or single serum samples as the species with the highest difference in titers or the highest titer. In case of equivalent titers for >1 species, we considered the results unidentified species of SFGR. We considered a single serum IgG titer of 1/64 a minimum positive titer, which supports *Rickettsiosis* for patients for surveillance purposes at CDC with a clinically compatible acute illness and with an epidemiologic history of the disease (*5*). However, to increase the specificity for identification of clinical cases, we included only patients with 1 serum sample with titers >1:128 (probable cases in our study) in the data analysis (*24*)*.*

### Sampling and Data Collection of Ticks

Two groups collected ticks for 4 days (May 13–16) in 2 districts of Pavlodar region. Using a map and a hand-held global positioning system tracker, we selected 4 ordinal points within each designated village for tick collection. Groups conducted sampling for >3 person-hours at each location within a suitable habitat (i.e., woods, grassland, and trail margins) (*25*).

We collected ticks using the dragging method, in which light-colored flannel material is dragged behind the collector by a rope handle attached to a wooden base. We used a flagging method in areas with high vegetation density in which the drag cloth could not be pulled between plants. For the flagging method, we used the material attached to 1 end of a wooden rod like a flag to sweep it through vegetation (*26*). We collected ticks in the second week of May 2019. This window coincided with peak activity for *Dermacentor* ticks, which are known to carry *Rickettsia* species (*27*–*29*).

We identified tick species, development stage, and sex morphologically using a stereomicroscope according to published tick specification guidelines (*30*,*31*). We then placed adult ticks individually into tubes containing 70% ethanol for storage before sending them to the Central Reference Laboratory for testing.

### Laboratory Testing of Ticks

We homogenized each tick specimen individually using the TissueLyser II (QIAGEN). We extracted total nucleic acid using the PrepMan Ultra Sample Preparation Reagent (ThermoFisher Scientific) according to the manufacturer’s instructions. We used *Rickettsia* genus-specific (pan-*Rickettsia*) real-time PCR for testing each tick individually. We tested positive samples using a seminested PCR for amplification and sequencing targeting r*Omp*A gene to determine species, as described previously for human samples.

### Key Definitions

We defined a suspected case of rickettsiosis as illness in a person hospitalized with fever (temperature >38°C) and >1 of the following clinical signs or symptoms: rash, eschar, headache, myalgia, anemia, thrombocytopenia, hepatic transaminase elevation (*16*), or suspicion of rickettsiosis by the clinician. We defined a probable case of rickettsiosis as illness in a person meeting the definition of a suspected case of rickettsiosis with only 1 serum sample available, with IFA *Rickettsia* IgG titers >1:128 (*24*). We defined a laboratory-confirmed case of rickettsiosis as illness in a person meeting the definition of a suspected case of rickettsiosis with a positive PCR result for blood, eschar, or skin, or a 4-fold increase in IFA *Rickettsia* IgG titers (*R. raoultii*, *R. sibirica*, or *R. slovaca*) from a sample taken upon admission and a convalescent sample (*16*). Severe disease was defined by clinicians as having any of the following: acute respiratory distress syndrome (shortness of breath [respiration rate >25 breaths/min], cyanosis, bilateral infiltrates on the lung radiograph, progressive hypoxemia [SpO_2_ ≤90%]); disseminated intravascular coagulation (blood clotting time >12 s, plasma D-dimer >250 µg/L, platelet count <120 × 10^9^/L, plasma fibrinogen <1.5 g/L, prothrombin time (PT) >16 s); or renal failure (oliguria up to anuria and accompanied by proteinuria of >0.5 g/L, serum creatinine >117 mkmol/L, blood serum urea >8 mmol/L).

### Data Analysis

Our data analysis only included laboratory-confirmed and probable cases. Electronic questionnaire development, data entry, data cleaning, and analysis were done in EpiInfo 7.2. We used the χ^2^ or Fisher exact tests to assess the differences in clinical symptoms by *Rickettsia* species. We considered a p value of <0.05 significant.

### Ethics Statement

This study was approved by the Ethical Committee of the Kazakh Medical University (“The Highest School of Public Health”) (protocol no. IRB-A096, April 2019) and extended (protocol no. IRB-A12, September 2020). This activity was reviewed by CDC, deemed not research, and was conducted consistent with applicable federal law and CDC policy (e.g., 45 C.F.R. part 46, 21 C.F.R. part 56; 42 U.S.C. §241(d); 5 U.S.C. §552a; 44 U.S.C. §3501 et seq.).

## Results

### Characteristics of Participants

We obtained consent from and enrolled 105 persons hospitalized with suspected rickettsiosis; among those, 62 (59%) were determined to be laboratory-confirmed or probable rickettsiosis. Among participants with rickettsiosis, 20 (32%) were confirmed by IFA in paired samples (a 4-fold increase of IgG titers to 1 of 3 investigated *Rickettsia* species), 35 (56%) by PCR, and 7 (11%) as probable cases in a single available acute sample with a titer of >1:128 by IFA. We did not include 10 patients with a titer of 1:64 with a single available IFA in the analysis. We identified 3 *Rickettsia* species among the participants: *R. sibirica* (47%), *R. raoultii* (31%), and *R. slovaca* (6%). No *Rickettsia*-specific species were identified in 16% of participants. (Appendix Table 1).

Of the 62 participants with acute rickettsiosis, 85% were adults (>18 years of age), 48% were male, 66% lived in rural areas or suburbs, 55% owned a cat or dog, and 23% owned livestock (Table 1). In the 2 weeks before disease onset, 53% had worked in a garden or orchard, 48% had noted a tick bite, and 31% had been in an open countryside area. Fifty-eight percent of participants reported usually removing ticks with their bare hands. To protect themselves against tick bites, 32% reported taking some protective measures, such as wearing long-sleeved clothing or gloves or using repellents; 68% reported taking no measures (Table 1).

**Table 1:** Demographic and tick-related behavior characteristics of 62 patients in study of spotted fever group rickettsioses among hospitalized patients and circulation of *Rickettsia* in ticks, Kazakhstan, 2019

Characteristic	No. (%)
Age group, y	
>18	53 (85)
<18	9 (15)
Sex	
M	30 (48)
F	32 (52)
Place of residence	
Countryside/suburb*	41 (66)
City	21 (34)
Owned a summer cottage	9 (15)
Owned pets (cats, dogs)	34 (55)
Owned livestock	14 (23)
During the previous 2 weeks	
Worked in a garden or orchard	33 (53)
Noticed a tick bite	30 (48)
Went out to the open countryside	19 (31)
Usual method of tick removal	
Removed ticks with bare hands	36 (58)
Removed ticks at the hospital or polyclinic	7 (11)
Using tweezers to remove tick	2 (3)
Using gloves when removing	1 (2)
Other methods of removing	3 (5)
Never removed ticks	13 (21)
Methods used to protect yourself from ticks	
None	42 (68)
Long clothes in several layers, gloves†	14 (23)
Repellents only	5 (8)
Avoided bushes	1 (2)
Methods used to protect your pets, n = 34‡	
None	30 (88)
Insecticides	4 (12)

*If a patient lived in a dacha (summer cottage) for >1 month, the place of residence was considered a suburb. †This number includes 4 patients who used the listed items and repellents. ‡Protection of pets was calculated for patients who had pets.

We detected rickettsiosis cases in all sentinel sites. Eleven percent of cases were primarily referrals from nonsentinel sites to the regional hospitals in Pavlodar City. Based on the place of residence, we identified rickettsioses cases in 85% (11/13) of the administrative territories of Pavlodar region.

Rickettsiosis cases peaked in May (53% of cases). Although *R. sibirica* cases occurred throughout the endemic season, *R. raoultii* cases occurred predominantly in the summer months (Appendix Figure).

### Clinical Features

The most common signs and symptoms among those with rickettsiosis were fever (100%), headache (90%), rash (79%), myalgia (61%), and eschar (48%). More than half (53%) had thrombocytopenia, and 39% had elevated aspartate aminotransferase. Fifteen percent had severe disease; no deaths occurred (Table 2).

**Table 2:** Characteristics of 62 human rickettsiosis cases by *Rickettsia* species in study of spotted fever group rickettsioses among hospitalized patients and circulation of *Rickettsia* in ticks, Kazakhstan, 2019*

Characteristic	Total	*R. sibirica *	*R. raoultii *	SFGR	*R. slovaca*
No. patients	62 (100)	29 (100)	19 (100)	10 (100)	4 (100)
Symptoms and signs					
Fever >38°C	62 (100)	29 (100)	19 (100)	10 (100)	4 (100)
Headache	56 (90)	27 (93)	17 (89)	9 (90)	3 (75)
** Rash**	**49 (79)**	**26 (90)**	**12 (63)**	**8 (80)**	**3 (75)**
Myalgia	38 (61)	21 (72)	11 (58)	5 (50)	1 (25)
** Primary skin lesion: eschar**	**30 (48)**	**23 (79)**	**2 (11)**	**4 (40)**	**1 (25)**
Nausea	18 (29)	8 (28)	7 (37)	3 (30)	0
Vomiting	11 (18)	5 (17)	4 (21)	2 (20)	0
** Swollen lymph nodes **	**3 (5)**	**1 (4)**	**1 (5)**	**0**	**1 (25)**
**Any rash, eschar, or swollen lymph nodes**†	**54 (87)**	**28 (97)**	**14 (74)**	**9 (90)**	**3 (75)**
Severe disease‡†					
Had severe disease	9 (15)	6 (21)	1 (5)	2 (20)	0
Had acute respiratory distress syndrome	6 (10)	5 (17)	1 (5)	0	0
Had disseminated intravascular coagulation	4 (6)	2 (7)	0	2 (20)	0
Had renal failure	2 (3)	2 (7)	0	0	0
Blood abnormalities					
Thrombocytopenia	33 (53)	18 (62)	12 (63)	3 (30)	0
AST elevation§	23 (39)	12 (46)	8 (44)	2 (20)	1 (25)
ALT elevation§	11 (19)	6 (23)	1 (6)	1 (10)	3 (75)
Anemia	6 (10)	3 (10)	1 (5)	2 (20)	0

*Values are no. (%). The 62 patients include 55 confirmed (35 by PCR and 20 by indirect immunofluorescence assay with 4-fold seroconversion) and 7 probable cases (IgG titers >1:128). Probable cases with the only available acute serum had IgG titers of 1:128. The species of *Rickettsia* implicated in the disease for patients with paired or single serum samples were defined in indirect immunofluorescence assay as the species with the highest difference in titers or the highest titer. Bold indicates cutaneous signs and lymphadenopathy (swollen lymph nodes). ALT, alanine aminotransferase; AST, aspartate aminotransferase; SFGR, spotted fever group rickettsia. †All patients with swollen lymph nodes had a rash or eschar. ‡Severe disease was diagnosed on the basis of the following criteria: 1) renal failure (oliguria up to anuria and accompanied by proteinuria of >0.5 g/L, serum creatinine >117 mkmol/L, blood serum urea >8 mmol/L); 2) acute respiratory distress syndrome (shortness of breath (respiration rate >25 breaths/min), cyanosis, bilateral infiltrates on the lung radiograph, progressive hypoxemia (SpO_2_ <90%); 3) disseminated intravascular coagulation (blood clotting time (>12 s), plasma D-dimer (>250 µg/l), platelet count (<120*10^9^/l), plasma fibrinogen (<1.5 g/L), prothrombin time (PT) (>16 s). Blood abnormalities assessment was done by clinicians using the following levels: thrombocytopenia, platelet (<150 × 10^9^/L); leukopenia, leukocytes <4 × 10^9^ cells/L); anemia, hemoglobin <130 g/L for men, <120 g/L for women, <115 g/L for children; AST elevation, >40 U/L for men, 31 U/L for women, >43 U/L for children; ALT elevation, >40 U/L for men, 33 U/L for women, >36 U/L for children). §Three *R. sibirica* cases had no data on AST or ALT.

Rash, eschar, or swollen lymph nodes were present in 87% and absent in 13% of patients. Eschar was the most common among participants with *R. sibirica* and least common among those with *R. raoultii* with a difference in prevalence of 7.5 times (crude prevalence ratio 7.5 [95% CI –2.0 to 28.3]; p<0.001). Severe disease was also most common among participants with *R. sibirica* (21%) and least common among those with *R. raoultii* (5%) or *R. slovaca* (0%). (Table 2).

### *Rickettsia* Species in *D. marginatus* and *D. reticulatus* Ticks

We collected 959 ticks; 64% were identified as *D. reticulatus* ticks and 36% were *D. marginatus* ticks. *R. raoultii* was detected in 46 (5%), SFG rickettsiae in 37 (4%), and *R. sibirica* in only 2 ticks. In both tick species, *R. raoultii* was in almost equivalent proportions of 4% (13) in *D. marginatus* ticks to 5% (33) in *D. reticulatus* ticks (Appendix Table 2).

## Discussion

*Rickettsioses* were detected in ticks or humans in 85% of districts in the Pavlodar region. By establishing sentinel sites and enhanced laboratory testing for surveillance of rickettsioses, we observed a 27% increase in the number of detected cases compared with the mean of the previous 3 years (62 vs. 49). *R. sibirica* and *R. raoultii* were the most frequent etiologic agents identified among cases. Those findings highlight missed opportunities for diagnosis and treatment of rickettsioses.

Our observation of a 27% increase in the number of detected cases compared with the mean of the previous 3 years after we established sentinel sites for surveillance is concerning because of potential gaps in medical interventions for persons with rickettsioses. Although most persons had mild illness, 15% had severe disease. Empirical treatment is most effective at preventing death and severe illness when started within the first 5 days after symptom onset. Although our findings do not enable us to evaluate missed opportunities for early treatment, the substantial underdetection of cases in an endemic region suggests there could be infected persons who develop severe disease or are at risk for developing severe disease who do not receive the appropriate diagnosis and early treatment by healthcare providers (*32*,*33*).

Consistent with known clinical manifestations of SFGR, fever, headache, and myalgia were the most common symptoms among case-patients (*5*). Cutaneous findings occurred frequently, but 13% of case-patients had none. Cutaneous signs can differ widely by species. The presence of eschar has been reported to be 7 times less common among persons with *R. raoultii* than *R. sibirica* (*5*,*34*,*35*), and we found that 26% of participants with *R. raoultii* did not have cutaneous signs. Because of the lack of a standard case definition and clinical protocol for rickettsioses in Kazakhstan, patients without cutaneous signs might not receive appropriate diagnostics or treatment for rickettsioses. The inclusion of headache, fever, or myalgia in the clinical case definition for suspected rickettsioses could contribute to improved recognition of the disease and faster initiation of appropriate treatment.

*D. marginatus* and *D. reticulatus* ticks were the only species identified in our study. The detection of *R. raoultii* and *R. sibirica* in those ticks suggests that they might serve as vectors of SFGR in the Pavlodar region. However, given that only 3%–5% of the tested ticks were positive for any *Rickettsia* species, this association should be interpreted with caution. The vector capacity of *D. marginatus* and *D. reticulatus* ticks in transmitting *R. raoultii* has been demonstrated in previous studies (*11*). Transmission of *R. sibirica* is generally associated with *Haemaphysalis concinna, Dermacentor* spp., and *Hyalomma* spp. ticks (*8*,*13*,*36*). *H. concinna* ticks have been found in Pavlodar region by a previous study (*37*). Therefore, tick species other than those identified in this study might also contribute to the transmission of *Rickettsia* in the region. This information can assist in prompting clinicians to suspect rickettsioses among persons who are bitten by ticks of those species. In addition, persons bitten by those ticks can be given information about the need to self-monitor for fever, headache, myalgia, eschar, or rash and to seek medical care if those signs develop.

We also identified gaps in the use of protective clothing and tick removal practices. Regular use of repellents and protective clothing that covers the skin are recommended to decrease the risk for tick bites when spending time outdoors in tick environments in rickettsioses-endemic regions (*38*,*39*). Public health risk communication about preventing tick exposure can help increase awareness of tickborne diseases and ways to reduce the risk for tick bites.

The first limitation of our study is that fever was a required symptom for participation, so cases of asymptomatic SFGR could have been missed. We also did not survey all hospitals in the region. We included the reference hospital where the most severe cases are transferred. Those limitations might have resulted in an underestimation of the total number of cases in the region but might also have resulted in an overestimation of cases of severe disease among persons with SFGR. PCR confirmation of the rickettsia species in our study was obtained on the basis of a short sequence of 1 gene (rOmpA), which is commonly used in *Rickettsia* diagnostics (*19*). However, relying on a single, short amplicon limits the confidence with which one can make a precise species diagnosis because of the high genetic similarity and conserved nature of *Rickettsia* genomes. We could not identify species of *Rickettsia* for 6 rickettsioses and 37 ticks (SFGR without species identification), which might have been related to the sequencing methodology of only using the short sequence of 1 gene. The methods used could possibly have contributed to misclassification or missed diagnoses of species. Sequencing of 2 or 3 genes would have improved the accuracy and reliability of species differentiation. Last, we collected ticks only from 2 districts, which could have resulted in incomplete identification of ticks and *Rickettsia* species diversity (*40*).

In conclusion, we identified rickettsia-positive humans in at least 85% of the Pavlodar region districts and positive ticks in all 2 investigated districts. The predominant species identified among persons hospitalized with rickettsioses were *R. sibirica* and *R. raoultii.* Among the collected ticks, *Rickettsia* prevalence was ≈9%, and 5% carried *R. raoultii*. Almost 80% of persons with *Rickettsia* had a rash, half had an eschar, and 13% had no SFGR cutaneous signs or lymphadenopathy. More than half of participants removed ticks with bare hands, and almost 70% reported not using any protection against tick bites. Enhanced routine testing in the region is key to improving clinical management and disease surveillance. Increased awareness among clinicians and the general population about rickettsial symptoms and risk for tick bites could help reduce disease transmission and improve medical care.

Appendix Additional information about spotted fever group rickettsioses among hospitalized patients and circulation of *Rickettsia* in ticks, Kazakhstan, 2019

## References

[1] Parola P, Paddock CD, Socolovschi C, Labruna MB, Mediannikov O, Kernif T, et al. Update on tick-borne rickettsioses around the world: a geographic approach. Clin Microbiol Rev. 2013;26:657–702. 10.1128/CMR.00032-13 24092850 PMC3811236

[2] Nicholson W, Paddock C. CDC Yellow Book [cited 2019 Feb 22]. https://wwwnc.cdc.gov/travel/yellowbook/2024/infections-diseases/Rickettsial-diseases/1000

[3] Botelho-Nevers E, Socolovschi C, Raoult D, Parola P. Treatment of *Rickettsia* spp. infections: a review. Expert Rev Anti Infect Ther. 2012;10:1425–37. 10.1586/eri.12.139 23253320

[4] Ogden NH, Ben Beard C, Ginsberg HS, Tsao JI. Possible effects of climate change on Ixodid ticks and the pathogens they transmit: predictions and observations. J Med Entomol. 2021;58:1536–45. 10.1093/jme/tjaa220 33112403 PMC9620468

[5] Biggs HM, Behravesh CB, Bradley KK, Dahlgren FS, Drexler NA, Dumler JS, et al. Diagnosis and management of tickborne rickettsial diseases: Rocky Mountain spotted fever and other spotted fever group rickettsioses, ehrlichiosis, and anaplasmosis—United States. MMWR Recomm Rep. 2016;65:1–44. 10.15585/mmwr.rr6502a1 27172113

[6] Satjanadumrong J, Robinson MT, Hughes T, Blacksell SD. Distribution and ecological drivers of spotted fever group Rickettsia in Asia. Ecohealth. 2019;16:611–26. 10.1007/s10393-019-01409-3 30993545 PMC6910891

[7] Georgiades K, Merhej V, Raoult D. The influence of rickettsiologists on post-modern microbiology. Front Cell Infect Microbiol. 2011;1:8. 10.3389/fcimb.2011.00008 22919574 PMC3417371

[8] Rudakov NV, Shpynov SN, Samoilenko IE, Tankibaev MA. Ecology and epidemiology of spotted fever group Rickettsiae and new data from their study in Russia and Kazakhstan. Ann N Y Acad Sci. 2003;990:12–24. 10.1111/j.1749-6632.2003.tb07332.x 12860595

[9] Shpynov SN, Parola P, Rudakov NV, Samoĭlenko IE, Tankibaev MA, Tarasevich IV, et al. [Genotyping of spotted tick fever rickettsiae, detected in Russia and Kazakhstan] [in Russian]. Med Parazitol (Mosk). 2003;3:20–4. 14564838

[10] Shpynov S, Fournier PE, Rudakov N, Tankibaev M, Tarasevich I, Raoult D. Detection of a rickettsia closely related to *Rickettsia aeschlimannii*, “*Rickettsia heilongjiangensis*,” *Rickettsia* sp. strain RpA4, and *Ehrlichia muris* in ticks collected in Russia and Kazakhstan. J Clin Microbiol. 2004;42:2221–3. 10.1128/JCM.42.5.2221-2223.2004 15131195 PMC404608

[11] Samoylenko I, Shpynov S, Raoult D, Rudakov N, Fournier PE. Evaluation of *Dermacen*tor species naturally infected with *Rickettsia raoultii.* Clin Microbiol Infect. 2009;15(Suppl 2):305–6. 10.1111/j.1469-0691.2008.02249.x 19438650

[12] Perfilyeva YV, Shapiyeva ZZ, Ostapchuk YO, Berdygulova ZA, Bissenbay AO, Kulemin MV, et al. Tick-borne pathogens and their vectors in Kazakhstan - A review. Ticks Tick Borne Dis. 2020;11:101498. 10.1016/j.ttbdis.2020.101498 32723625

[13] Rudakov N, Shpynov S, Fournier PE, Raoult D. Ecology and molecular epidemiology of tick-borne rickettsioses and anaplasmoses with natural foci in Russia and Kazakhstan. Ann N Y Acad Sci. 2006;1078:299–304. 10.1196/annals.1374.009 17114725

[14] Yakubovskij VI, Igolkina YP, Tikunov AY, Panov VV, Yakymenko VV, Zhabykpayeva AG, et al. Genetic diversity of rickettsiae in *Dermacentor* spp. ticks on the territory of western Siberia and northern Kazakhstan. Mol Gen Microbiol Virol. 2023;38:158–67. 10.3103/S0891416823030102

[15] Turebekov N, Abdiyeva K, Yegemberdiyeva R, Dmitrovsky A, Yeraliyeva L, Shapiyeva Z, et al. Prevalence of *Rickettsia* species in ticks including identification of unknown species in two regions in Kazakhstan. Parasit Vectors. 2019;12:197. 10.1186/s13071-019-3440-9 31053085 PMC6500025

[16] Centers for Disease Control and Prevention. Spotted fever rickettsiosis (*Rickettsia* spp.) 2010 case definition [cited 2019 Feb 22]. https://wwwn.cdc.gov/nndss/conditions/spotted-fever-rickettsiosis/case-definition/2010/

[17] Centers for Disease Control and Prevention. Specimen submission guidelines. Skin biopsy specimens [cited 2019 Feb 22]. https://www.cdc.gov/ncezid/dvbd/pdf/collection-submission-skin-biopsy-specimens-rickettsial-disease.pdf

[18] Centers for Disease Control and Prevention. Collecting a eschar swab for rickettsial diagnostics [cited 2019 Feb 22]. https://www.cdc.gov/vector-borne-diseases/media/pdfs/2024/06/FS_Collection-Submission-Eschar-Swab-Specimens-Rickettsial-Disease-508.pdf

[19] Kato CY, Chung IH, Robinson LK, Austin AL, Dasch GA, Massung RF. Assessment of real-time PCR assay for detection of *Rickettsia* spp. and *Rickettsia rickettsii* in banked clinical samples. J Clin Microbiol. 2013;51:314–7. 10.1128/JCM.01723-12 23135935 PMC3536194

[20] Roux V, Fournier PE, Raoult D. Differentiation of spotted fever group rickettsiae by sequencing and analysis of restriction fragment length polymorphism of PCR-amplified DNA of the gene encoding the protein rOmpA. J Clin Microbiol. 1996;34:2058–65. 10.1128/jcm.34.9.2058-2065.1996 8862558 PMC229190

[21] Eremeeva ME, Liang Z, Paddock C, Zaki S, Vandenbergh JG, Dasch GA, et al. *Rickettsia rickettsii* infection in the pine vole, *Microtus pinetorum*: kinetics of infection and quantitation of antioxidant enzyme gene expression by RT-PCR. Ann N Y Acad Sci. 2003;990:468–73. 10.1111/j.1749-6632.2003.tb07412.x 12860675

[22] Edgar RC. MUSCLE: multiple sequence alignment with high accuracy and high throughput. Nucleic Acids Res. 2004;32:1792–7. 10.1093/nar/gkh340 15034147 PMC390337

[23] Brouqui P, Bacellar F, Baranton G, Birtles RJ, Bjoërsdorff A, Blanco JR, et al.; ESCMID Study Group on Coxiella, Anaplasma, Rickettsia and Bartonella; European Network for Surveillance of Tick-Borne Diseases. Guidelines for the diagnosis of tick-borne bacterial diseases in Europe. Clin Microbiol Infect. 2004;10:1108–32. 10.1111/j.1469-0691.2004.01019.x 15606643

[24] Centers for Disease Control and Prevention. Spotted fever rickettsiosis (including Rocky mountain spotted fever) (SFR, including RMSF) 2020 case definition [cited 2025 Apr 19]. https://ndc.services.cdc.gov/case-definitions/spotted-fever-rickettsiosis-2020

[25] Rulison EL, Kuczaj I, Pang G, Hickling GJ, Tsao JI, Ginsberg HS. Flagging versus dragging as sampling methods for nymphal *Ixodes scapularis* (Acari: Ixodidae). J Vector Ecol. 2013;38:163–7. 10.1111/j.1948-7134.2013.12022.x 23701621

[26] Ontario Agency for Health Protection and Promotion (Public Health Ontario). Tick dragging: standard operating procedure. Toronto: Queen’s Printer for Ontario; 2015.

[27] Obert AS, Kurepina NY, Bezrukov GV, Merkushev OA, Cherkashina EN, Kalinina UV. Ixodid ticks as carriers of human transmissible infectious diseases in Altai Krai. J Altai Branch Russ Geogr Soc. 2015;37:82–9.

[28] Földvári G, Široký P, Szekeres S, Majoros G, Sprong H. *Dermacentor reticulatus*: a vector on the rise. Parasit Vectors. 2016;9:314. 10.1186/s13071-016-1599-x 27251148 PMC4888597

[29] Fan MY, Walker DH, Yu SR, Liu QH. Epidemiology and ecology of rickettsial diseases in the People’s Republic of China. Rev Infect Dis. 1987;9:823–40. 10.1093/clinids/9.4.823 3326129

[30] Filippova NA. Ixodid ticks of the *Amblyomminae* subfamily. Fauna of Russia and neighboring countries [in Russian]. In: Arachnids. St. Petersburg (Russia): Nauka; 1977.

[31] Guglielmone AA, Robbins RG, Apanaskevich DA, Petney TN, Estrada-Peña A, Horak IG, et al. The *Argasidae, Ixodidae* and *Nuttalliellidae* (*Acari:* Ixodida) of the world: a list of valid species names. Zootaxa. 2010;2528:1–28. 10.11646/zootaxa.2528.1.1

[32] Kirkland KB, Wilkinson WE, Sexton DJ. Therapeutic delay and mortality in cases of Rocky Mountain spotted fever. Clin Infect Dis. 1995;20:1118–21. 10.1093/clinids/20.5.1118 7619985

[33] Blanton LS. The rickettsioses: a practical update. Infect Dis Clin North Am. 2019;33:213–29. 10.1016/j.idc.2018.10.010 30712763 PMC6364315

[34] Li H, Zhang PH, Huang Y, Du J, Cui N, Yang ZD, et al. Isolation and identification of *Rickettsia raoultii* in human cases: a surveillance study in 3 medical centers in China. Clin Infect Dis. 2018;66:1109–15. 10.1093/cid/cix917 29069294

[35] Igolkina Y, Rar V, Krasnova E, Filimonova E, Tikunov A, Epikhina T, et al. Occurrence and clinical manifestations of tick-borne rickettsioses in Western Siberia: First Russian cases of *Rickettsia aeschlimannii* and *Rickettsia slovaca* infections. Ticks Tick Borne Dis. 2022;13:101927. 10.1016/j.ttbdis.2022.101927 35220061

[36] Mediannikov OY, Sidelnikov Y, Ivanov L, Mokretsova E, Fournier PE, Tarasevich I, et al. Acute tick-borne rickettsiosis caused by *Rickettsia heilongjiangensis* in Russian Far East. Emerg Infect Dis. 2004;10:810–7. 10.3201/eid1005.030437 15200813 PMC3323216

[37] Amirova NA, Pakizh VI, Chepeljuk MA, Suprun VG, Sergeeva NI, Tilik AN. Ixodid ticks from the Pavlodar district and their participation in the tularemia infection circulation. Parazitologiia. 1989;23:267–74. 2528108

[38] Vaughn MF, Funkhouser SW, Lin FC, Fine J, Juliano JJ, Apperson CS, et al. Long-lasting permethrin impregnated uniforms: A randomized-controlled trial for tick bite prevention. Am J Prev Med. 2014;46:473–80. 10.1016/j.amepre.2014.01.008 24745637

[39] Eisen L. Personal protection measures to prevent tick bites in the United States: Knowledge gaps, challenges, and opportunities. Ticks Tick Borne Dis. 2022;13:101944. 10.1016/j.ttbdis.2022.101944 35364518 PMC10859966

[40] Černý J, Buyannemekh B, Needham T, Gankhuyag G, Oyuntsetseg D. Hard ticks and tick-borne pathogens in Mongolia-A review. Ticks Tick Borne Dis. 2019;10:101268. 10.1016/j.ttbdis.2019.101268 31471272

