# Supplementary material for: Spotted Fever Group Rickettsioses among Hospitalized Patients and Circulation of Rickettsia in Ticks, Kazakhstan, 2019
Source: Emerg Infect Dis. 2025 Oct;31(10):1961–8. doi: 10.3201/eid3110.250037 (PMC12483003; doi:10.3201/eid3110.250037)
Supplement: Appendix — Additional information about spotted fever group rickettsioses among hospitalized patients and circulation of Rickettsia in ticks, Kazakhstan, 2019 [file 25-0037-Techapp-s1.pdf]

Article DOI: https://doi.org/10.3201/eid3110.250037

*EID cannot ensure accessibility for supplementary materials supplied by authors. Readers who have difficulty accessing supplementary content should contact the authors for assistance.*

# Spotted Fever Group Rickettsioses among Hospitalized Patients and Circulation of *Rickettsia* in Ticks, Kazakhstan, 2019

## Appendix

In a cross-sectional study of the identification of rickettsioses among hospitalized patients in Pavlodar region during May-October 2019, we found 62 rickettsia cases. A well-marked peak in rickettsioses incidence was observed in May (53% out of all cases) when all identified species were presented. In August-September, cases caused by *R. raoultii* were not identified in contrast to *R. sibirica* which presented from May to September.

**Appendix Table 1.** *Rickettsia* species detected in human rickettsioses by testing types, six sentinel sites of Pavlodar region, 2019 (n = 62)*

| Characteristic | No. (%) |
|---|---|
| Species | |
| *R. sibirica* | 29 (47) |
| *R. raoultii* | 19 (31) |
| SFGR | 10 (16) |
| *R. slovaca* | 4 (6) |
| Method of detection | |
| PCR | 35 (56) |
| *R. sibirica* | *28 (80)* |
| *SFGR* | *6 (17)* |
| *R. slovaca* | *1(3)* |
| IFA with paired serums *(4-fold increase)* | 20 (32) |
| *R. raoultii* | *13 (65)* |
| *R. slovaca* | *3 (15)* |
| *SFGR* | *3 (15)* |
| *R. sibirica* | *1 (5)* |
| IFA with only a single serum collected *(titers ≥1/128)* | 7 (11) |
| *R. raoultii* | *6 (86)* |
| *SFGR* | *1 (14)* |

*IFA, immunofluorescence assay; SFGR, Spotted fever group rickettsia (without species identification). The species of *Rickettsia* in IFA with paired or single sera were defined as the species with the highest difference in titers or the highest titer. In a cross-sectional study of the identification of rickettsioses among hospitalized patients in Pavlodar region, we found 62 rickettsioses. Ten rickettsioses with a titer of 1/64 (9 *R. raoultii* and 1 *R. slovaca*) were not included in the analysis to increase specificity. PCR assays were done only for patients with rash or eschar specimens (not blood). Second serums were collected in patients whose rash or eschar specimens were negative in PCR or who did not have a rash or eschar. The nucleotide sequences of the ompA gene of *Rickettsia spp*. identified among human samples have been deposited using a web-based submission tool BankIt, in the GenBank database under accession numbers (PQ511952-PQ511984). For four patients the sequences were obtained from two sources: skin biopsy and eschar. We found three species of *Rickettsia* but almost 80% of diseases were caused by *R. sibirica or R raoultii.*

**Appendix Table 2.** *Rickettsia* species in ticks (n = 959), Pavlodar and Aksu districts of Pavlodar region, May 2019*

| Tick characteristics | No. (%) |
|---|---|
| Tick species | |
| *Dermacentor reticulatus* | 610 (64) |
| *D. marginatus* | 349 (36) |
| Tick sex | |
| F | 632 (66) |
| M | 327 (34) |
| *Rickettsia* species in ticks | |
| *Rickettsia raoultii* | 46 (5) |
| *SFGR* | 37 (4) |
| *R. sibirica* | 2 (0) |
| *Rickettsia* species in *D. reticulatus* (n = 610) | |
| *R. raoultii* | 33 (5) |
| *SFGR* | 26 (4) |
| *R. sibirica* | 0 (0) |
| *Rickettsia* species in *D. marginatus* (n = 349) | |
| *R. raoultii* | 13 (4) |
| *SFGR* | 11 (3) |
| *R. sibirica* | 2 (0) |

**SFGR,* Spotted fever group rickettsia (without species identification). 959 ticks were collected from two districts of Pavlodar region in May 2019. Of them, two tick species were found. The proportion of *D. reticulatus* was almost twice as high as that *D. marginatus*. Each tick was tested individually by *Rickettsia* genus-specific (panRickettsia) real-time PCR assays followed by sequencing. The nucleotide sequences of the ompA gene of Rickettsia spp. identified among collected ticks have been deposited, using a web-based submission tool BankIt, in the GenBank database under accession numbers (PQ511762-PQ511809). The proportion of rickettsia-positive ticks was 9%, of which more than half were *R. raoultii*. In both tick species, *R. raoultii* was the most prevalent rickettsia identified among collected ticks. *R. sibirica* was found only in 2 ticks of *D. marginatus*.

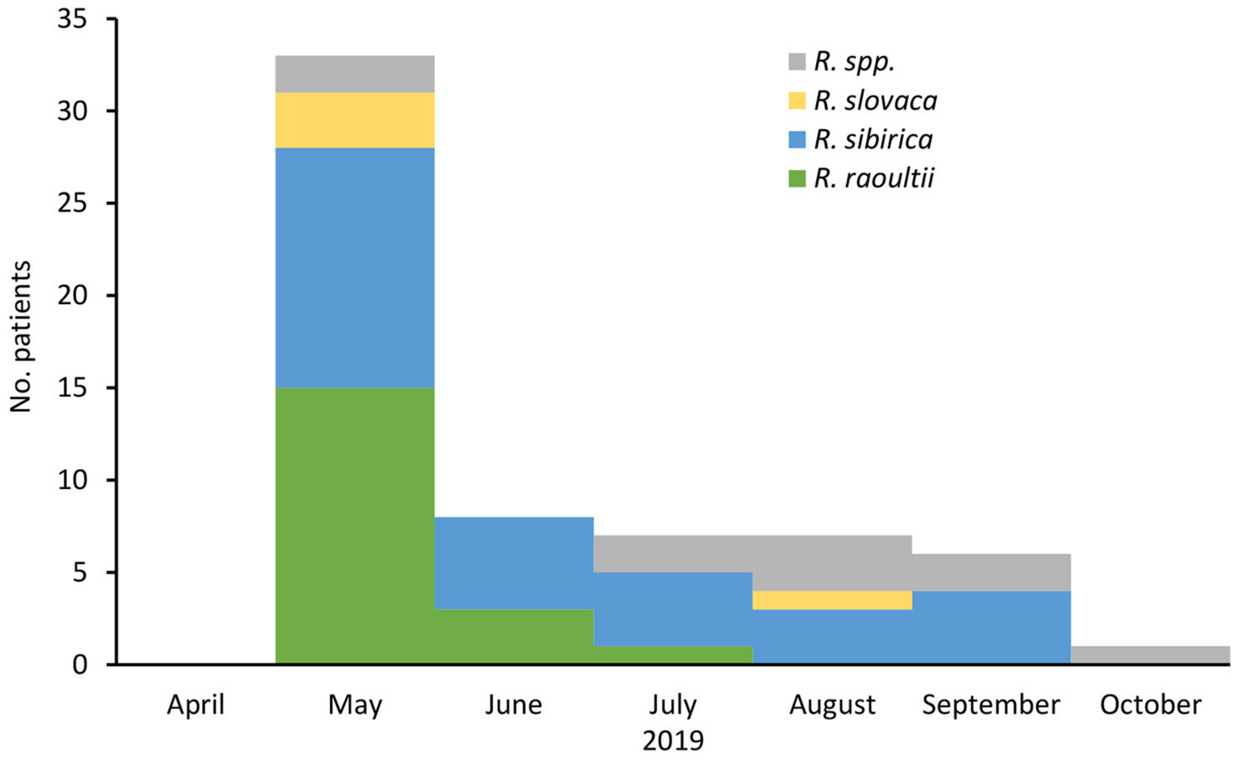


**Appendix Figure.** Human rickettsioses by species and month of disease onset, 2019, Pavlodar region of Kazakhstan (N = 62). *R. spp.*, spotted fever group rickettsia (without species identification).
